# Genome-Wide Identification and Characterization of the TCP Gene Family in Cucumber (*Cucumis sativus L.*) and Their Transcriptional Responses to Different Treatments

**DOI:** 10.3390/genes11111379

**Published:** 2020-11-20

**Authors:** Haifan Wen, Yue Chen, Hui Du, Leyu Zhang, Keyan Zhang, Huanle He, Junsong Pan, Run Cai, Gang Wang

**Affiliations:** 1 School of Agriculture and Biology, Shanghai Jiao Tong University, Shanghai 200240, China; haifanwen@sjtu.edu.cn (H.W.); yuechen321@sjtu.edu.cn (Y.C.); duhui1122@sjtu.edu.cn (H.D.); zly_sjtu@sjtu.edu.cn (L.Z.); lnykzky2016@sjtu.edu.cn (K.Z.); hlhe75@sjtu.edu.cn (H.H.); jspan71@sjtu.edu.cn (J.P.); cairun@sjtu.edu.cn (R.C.); 2 State Key Laboratory of Vegetable Germplasm Innovation, Tianjin 300384, China

**Keywords:** cucumber, expression pattern, *TCP* genes, organ development

## Abstract

TCP proteins are plant-specific transcription factors widely implicated in leaf morphogenesis and senescence, flowering, lateral branching, hormone crosstalk, and stress responses. However, the relationship between the transcription pattern of *TCPs* and organ development in cucumber has not been systematically studied. In this study, we performed a genome-wide identification of putative *TCP* genes and analyzed their chromosomal location, gene structure, conserved motif, and transcript expression. A total of 27 putative *TCP* genes were identified and characterized in cucumber. All 27 putative *CsTCP* genes were classified into class I and class II. Class I comprised 12 *CsTCPs* and Class II contained 15 *CsTCPs*. The 27 putative *CsTCP* genes were randomly distributed in five of seven chromosomes in cucumber. Four putative *CsTCP* genes were found to contain putative miR319 target sites. Quantitative RT-PCR revealed that 27 putative *CsTCP* genes exhibited different expression patterns in cucumber tissues and floral organ development. Transcript expression and phenotype analysis showed that the putative *CsTCP* genes responded to temperature and photoperiod and were induced by gibberellin (GA)and ethylene treatment, which suggested that *CsTCP* genes may regulate the lateral branching by involving in multiple signal pathways. These results lay the foundation for studying the function of cucumber *TCP* genes in the future.

## 1. Introduction

Transcription factors play significant roles in plant growth development. The *TCP* gene family is a plant-specific transcription factor family [1]. The name of *TCP* originated from the first four characterized members: TB1 (TEOSINTE BRANCHED 1) in *Zea mays* [2], CYC (CYCLOIDEA) in *Antirrhinum majus* [3], and the PCF1 and PCF2 (PROLIFERATING CELL FACTORS 1 and 2) in *Oryza sativa* [4]. The TCP transcription factors contain a conserved TCP domain, which constitutes a basic helix-loop-helix (bHLH) structure at the N-terminus. This domain is important for DNA binding and involved in protein–protein interactions [5].

According to the amino acid sequence similarity of the TCP domain, TCP factors were divided into two major classes: class I (PCF or TCP-P class) and class II (TCP-C class) [5,6]. The most significant difference between these two classes is a four-amino acid deletion in the basic domain of class I relative to class II proteins. The class II group can be further divided into subclasses, CIN and CYC/TB1, mainly based on differences in amino acid sequence, especially in the basic region of the TCP domain. Most members of the CYC/TB1 subclass containing an 18–20-residue arginine-rich motif (R domain), but little members of the CIN subclass contain the R domain [6,7,8]. Up to now, many *TCP* genes have been confirmed in various plant species. In silico studies on *TCPs* have identified 24 *TCPs* in *Arabidopsis*, 28 *TCPs* in *O. sativa* [8,9,10], 38 *TCPs* in *Gossypium raimondii* [11], and 23 *TCPs* in *Phalaenopsis equestris* [12]. *TCP* and their homologs are regulated by the conserved microRNA miR319 in different species. The miR319-regulated *TCPs* are involved in regulating the leaf morphogenesis [13], flower architecture [14], hormone biosynthesis and response [15]. In *Arabidopsis*, *AtTCP2-4*, *AtTCP10*, and *AtTCP24* are belong to the CIN family members which contain miR319 binding site.

Recent studies have shown that *TCPs* play crucial roles in plant growth and development. Several *TCPs* are implicated in trichome formation [16], gametophyte development [17], seed germination [18], floral development [19], and lateral shoot initiation [20]. Moreover, *TCPs* are also regulated by multiple hormone and external environmental signals. *GhTCP14* is a crucial regulator in auxin-mediated elongation of cotton fiber cells [16]. In *Arabidopsis*, *TCP15* is induced by gibberellins (GAs) and *TCP1* is a positive regulator of the brassinosteroid (BR) biosynthesis pathway [21,22]. Besides, *TCPs* also participate in ethylene, strigolactone (SL) and cytokinins (CK) biosynthesis [20,23]. *TCP20* and *TCP22* have a positive effect on regulating the circadian clock, and *TCP15* is induced by elevated temperature in *Arabidopsis* [24].

Cucumber (*Cucumis sativus* L.) is one of the most important vegetable crops worldwide [25]. Because of the diversity of flower sexual types, cucumber has become a model plant for researching the sex differentiation mechanism [26]. Despite the function of *TCP* genes are important, only a few cucumber *TCPs* were reported. *TEN*(*CsTCP21*), a *TCP* from cucumber, regulates the identity of tendrils [27,28]. *CsBRC1*(*CsTCP3*) inhibits lateral bud outgrowth by controlling auxin accumulation in axillary buds in cucumber [29]. Based on the first version of the cucumber genome, Yuan et al. identified 22 *TCPs* and performed bioinformatics analysis on them [30].

In this study, according to the second version of the cucumber genome with more detailed annotations, we identified 27 putative *TCP* genes and analyzed their chromosomal distribution, gene structure, conserved motif, phylogenetic relationship, and cis-acting elements on the promotors. We further analyzed *TCPs* expression profiles in different organs and response to the hormone treatment and environmental stresses in cucumber. The results should lay the foundation for studying the function of *CsTCPs* in the future.

## 2. Materials and Methods

### 2.1. Plant Materials, Growth Conditions, and Treatment

The cucumber monecious line 9930 was used to analyze gene expression patterns. Seeds were germinated on a wet filter paper in a Petri dish at 28 °C in the dark overnight. The seedings were then grown in a growth room of Shanghai Jiao Tong University (Shanghai, China) for 16 h/8 h at 25 °C/18 °C (day/night). At the two true leaf stage, the cucumber seedlings were used for exogenous hormone treatment. The apical shoots of seedings were soaked in water containing 100 μM gibberellin (GA) and 10 μM ethylene with 0.1% (*v/v*) Tween-20 for 2 h, respectively. The treatment with water containing 0.1% (*v/v*) Tween-20 was the control. After soaking, the apical shoots of seedings were sampled at 12 and 24 h, respectively. Some treated seedings were used for phenotype observation.

From sowing to the fourth true leaf unfolding, some seedings were grown in plant growth chambers under four different treatments, including low temperature (23 °C/15 °C, day/night) and short day (8 h/16 h day/night) (LS), low temperature and long day (16 h/8 h day/night) (LL), high temperature (32 °C/24 °C, day/night) and short day (HS), and high temperature and long day (HL). The treatment under high temperature with a photoperiod of 16 h/8 h (day/night) (HL) was a normal condition, considered to be the control. In addition, all treatments were repeated at least three times, and there were at least 15 seedings for each treatment. All materials were harvested and frozen immediately in liquid nitrogen and kept at −80 °C for RNA isolation.

### 2.2. Identification of TCP Genes in Cucumber

The whole sequence data of cucumber were obtained from the Cucurbit Genomics Database (CuGenDB; ftp://cucurbitgenomics.org/pub/cucurbit/genome/cucumber/Chinese_long/v2/). The TCP sequences of 24 *Arabidopsis* TCPs were retrieved from the Arabidopsis Information Resource (TAIR; https://www.arabidopsis.org/index.jsp). Two-step BLAST method was adopted to identify cucumber TCP genes. First, *Arabidopsis* TCPs were used to search possible cucumber TCPs with TBtools (e-value, 1e-5) [31]. Subsequently, all possible cucumber TCPs were further identified using National Center for Biotechnology Information (NCBI; https://www.ncbi.nlm.nih.gov/) BLASTP (e-value, 1e-5). Finally, candidate proteins were confirmed with SMART (http://smart.embl.de/) [32] and Pfam databases (http://pfam.xfam.org/) [33].

### 2.3. Phylogenetic Tree and Cis-Acting Elements Analysis

TCP protein sequences of 27 CsTCPs and 24 AtTCPs were used for phylogenetic analysis. The phylogenetic tree was constructed through the neighbor-joining (NJ) method using MEGAX software v.10.1.8 (https://www.megasoftware.net/) and the bootstraps test was carried out with 1000 iterations. The results were formatted for display using the Evolview V3 (https://www.evolgenius.info//evolview/#login) [34]. The gene structure of cucumber TCP genes was identified via TBtools. The online Multiple Expectation Maximization for Motif Elicitation (MEME) program version 5.0.5 (http://meme-suite.org/tools/meme) was used to predicted conserved motifs of the cucumber TCPs [35]. Finally, 2000 bp sequences upstream of the start codon ATG of each *TCP* gene was used to analyze the *cis*-acting elements using PlantCARE (http://bioinformatics.psb.ugent.be/webtools/plantcare/html/).

### 2.4. Analysis of Chromosomal Location and Collinearity Relationship and Prediction of miR319 Target Genes

The distribution information of cucumber *TCP* genes on the chromosomes was acquired using TBtools. Gene Collinearity analysis of *TCP* genes in different species was performed using the Multiple Collinearity Scan toolkit (MCScanX) with default parameters [36]. We plotted the collinearity relationship of the *TCP* genes from selected species using the TBtools software. Finally, psRNATarget (http://plantgrn.noble.org/psRNATarget/) was used to identify miR319 target genes by analyzing the full length of 27 *CsTCPs*.

### 2.5. Transcript Expression Analysis of TCP Genes

To analyze the *TCP* genes expression in different organs of cucumber, we sampled 11 organs of cucumber: root (3 week old seedings), stems (12 week old cucumber plants), leaf (3 week old seedings), cotyledon (3 week old seedings), tendril (12 week old cucumber plants), petal, stamen (1 day before flowering), pistil (1 day before flowering), carpel (1 day before flowering), pericarp (1 day before flowering) and trichome (1 day before flowering). In addition, the expression of 27 *CsTCPs* in flower buds at different development stages were also examined using real-time quantitative reverse transcription PCR (real-time qRT-PCR). The gene expression pattern analysis in different flower bud development periods was taken from the buds of different lengths, 1, 2, 5 and 10 mm.

Total RNA was extracted using OminiPlant RNA Kit (CWBIO, Beijing, China). The first-strand cDNA was synthesized using HiFiScript cDNA Synthesis Kit (CWBIO). Real-time qRT-PCR with UltraSYBR Mixture (CWBIO) was performed according to the manufacturers’ protocol. *CsActin3 (Csa6G484600.1)* was used as the internal control. All the qRT-PCR primers were designed using the Genious software according to the cDNA sequences (Tables S1 and S2). The 2^−ΔΔCt^ method was used to analyze the relative mRNA expression [37]. Each expression was repeated for three times biologically and technically under identical conditions.

## 3. Results

### 3.1. Identification and Chromosomal Distributions Analysis of Putative TCP Genes in Cucumber

We used a two-step BLAST method to identify *CsTCPs* from cucumber genome using the *TCPs* sequences of *Arabidopsis*. In total, 27 candidate *CsTCPs* were screened out, all of which were verified the existence of the TCP domains with the SMART and Pfam databases. The SMART analysis revealed that all putative cucumber TCP proteins contained the TCP domain, implying that there are at least 27 putative *TCP* genes in cucumber. The 27 putative *TCPs* were named *CsTCP1* to *CsTCP27* according to their accession number. These TCPs varied in their coding sequence (CDS), amino acid sequence, isoelectric point (pI), and molecular weight (MW) (Table S2). From the 27 CsTCPs, CsTCP18 is the smallest protein with 174 amino acids, whereas CsTCP27 is the largest protein with 449 amino acids. The protein MWs ranged from 18.91 kDa (CsTCP18) to 164.89 kDa (CsTCP27), and the pI value varied from 5.57 (CsTCP24) to 9.92 (CsTCP15). The 27 putative *CsTCP* genes are distributed on chromosome (Chr) 1, 3, 4, 5 and 7 (Figure S1). Among them, Chr1 contained the highest number of putative *TCP* genes, while no putative *TCP* genes were found on Chr2 and Chr7.

### 3.2. Phylogenetic Analysis and Classification Putative TCP Genes in Cucumber

**Figure 1:**
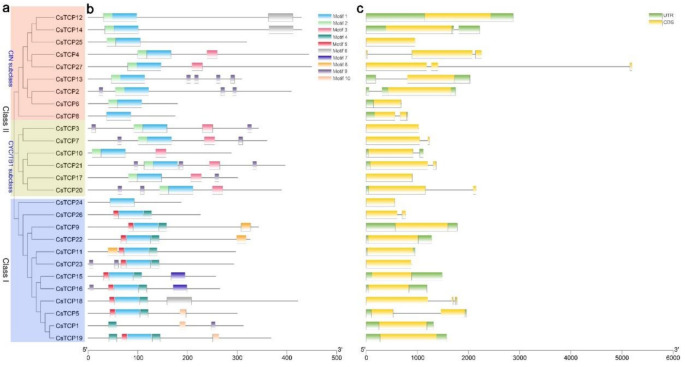
Motif composition and gene structure of cucumber putative *TCP* genes. (**a**). Phylogenetic tree of 27 cucumber TCPs. A specific color indicates each of the three TCP classes. (**b**). Motif composition of TCPs. Conserved motifs in the CsTCPs are shown in different colored boxes. (**c**). Gene structure analysis of putative *TCP* genes. Coding sequences (CDS), untranslated region (UTRs) and introns are represented by yellow boxes, green boxes, and black lines, respectively.

We constructed an unrooted phylogenetic tree using the neighbor-joining method (Figure S2, Figure 1a). The 51 TCPs from Arabidopsis and cucumber were divided into two classes, Class I (blue) and Class II, Class II could be further classified into two subclasses, CYC/TB1 and CIN. All *Arabidopsis* TCPs fell in the same Class as previously reported (Figure S2) [9]. There were 12 CsTCPs in class I. In the CYC/TB1 and CIN subgroup, there were six and nine CsTCPs, respectively (Figure S2, Figure 1a).

### 3.3. TCP Gene Structures and Conserved Motifs

According to the genome sequences and corresponding coding sequences of *TCP*s in cucumber, we found that the genome sequence lengths of *CsTCP* ranged from 561 to 5195 bp, and the lengths of CDS ranged from 525 to 1350 bp. The number of introns of these genes varies from zero to two. Two-thirds of *CsTCPs* contain only one exon and the other genes contain two or three exons, which is similar to the structure of the TCP family in other species genomes [11,38,39].

Ten conserved motifs labeled Motif 1 to Motif 10 were found in CsTCPs (Figure 1b). Motif 1 was annotated as the conserved bHLH structure, and presented in almost CsTCPs except CsTCP1, suggesting that it might be necessary for the CsTCPs to serve their function. Motifs 4, 7, 8 and 10 were only presented in Class I, while Motifs 2 and 3 were only presented in Class II. Thus, CsTCPs in the same class had similar motif composition while divergence was found in two class, indicated that CsTCPs in same class may perform the similar function and that some of motifs may play a vital role in specific function.

### 3.4. Collinearity Analysis of the Relationship among Cucumber, Melon (Cucumis melon) and Arabidopsis Members

**Figure 2:**
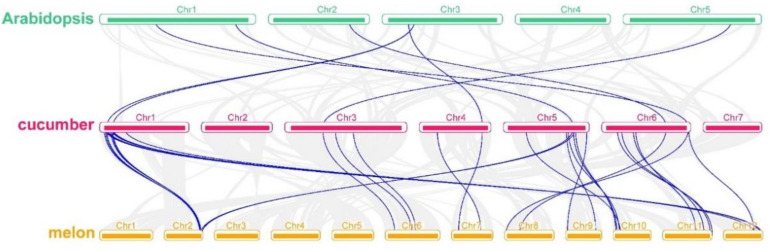
Collinear relationships of genes pairs from cucumber, melon and *Arabidopsis*. Blue lines indicate the collinear *TCP* gene pairs.

Combined analysis of *TCPs* from cucumber, melon and *Arabidopsis* was performed to study the gene collinear relationship among them (Figure 2). Collinearity analysis indicated that only seven (25.9%) collinear gene pairs within cucumber and *Arabidopsis* genomes. There was no gene on *Arabidopsis* Chr4 that was collinear with any putative *TCP* genes in cucumber genome. A total of 24 (88.9%) pairs of putative *TCP* genes were collinear between cucumber and melon (Table S3). These results indicated that putative *TCP* genes in cucumber and melon are highly conservative in evolution.

Based on the genome sequence analysis between cucumber and melon [40], cucumber Chr 1, 2, 3, 5 and 6 were collinear with melon Chr 2 and 12, 3 and 5, 4 and 6, 9 and 10, and 8 and 11, respectively. A few putative *TCP* genes does not conform to this correspondence. For example, cucumber *Csa5G605000* (Chr5) and *Csa5G608320.1* (Chr5) were collinear to melon *MELO3C017168.2.1* (Chr2) and *MELO3C017286.2.1* (Chr2), respectively. *Csa6G524000.1* (Chr6) was collinear to *MELO3C007121.2.1* on Chr 11 and *MELO3C002754.2.1* on Chr12 simultaneously. These results implied that the function of these genes may have diverged in cucumber and melon during evolution.

**Figure 3:**
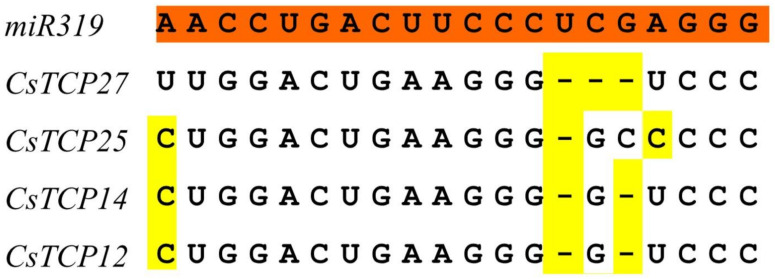
Alignment of putative target areas for miR319. Mismatches were represented by yellow.

In cucumber, the closest evolutionary *TCPs* with these genes are *CsTCP27*, *CsTCP25*, *CsTCP14* and *CsTCP12*, which contain sequences well matched with miR139 in the coding regions and might be the targets of miR319 (Figure 3). The other *CsTCPs* did not contain putative miR319 recognition site. These results indicated that the miR319 target sequence were conserved during the evolution of plants.

### 3.5. Promotor Cis-Acting Element Analysis of Putative CsTCP Genes

**Figure 4:**
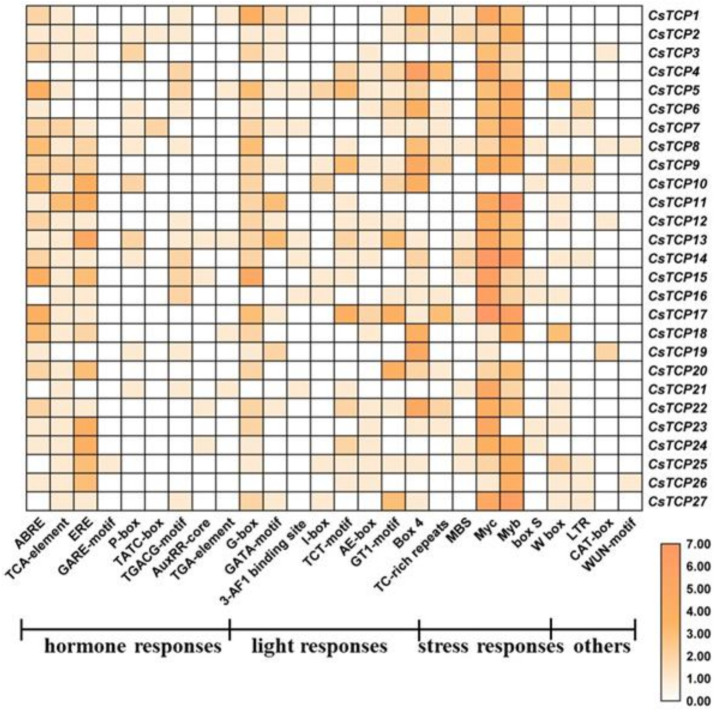
Cis-acting elements on promotors of *CsTCP*s. The color bar shows the number of cis-acting elements.

The *cis*-acting elements in the promoter of gene usually regulate gene expression pattern and location. In this study, a series of *cis*-acting elements involved in hormone response elements, light response elements, defense and stress response elements, low temperature wound response, and meristem expression were identified in putative *CsTCP* genes promotors (Figure 4 and Table S4).

For hormone-related *cis*-acting elements, we found abscisic acid (ABA) response elements (ABREs) and at least two or more ABRE *cis*-acting elements in the *CsTCPs* promotors, except *CsTCP4*, *CsTCP16*, *CsTCP25*, *CsTCP26* and *CsTCP27*. The salicylic acid (SA) response elements (TCA element) are relatively more extensively distributed, except *CsTCP4*, *CsTCP6* and *CsTCP19*. The ethylene response element (ERE) were found in three-quarters of these promotors. We also identified other hormone-related *cis*-acting elements, such as auxin response elements AuxRP and TGA elements, GA response elements GAREs, P-box and TATC-box, and MeJA response elements CGTCA and TGACG motifs in the promotors of some *CsTCPs* (Figure 4 and Table S4).

Additionally, we identified plenty of *cis*-acting elements related to light response in these promotors, including G-Box, GAG-motif, I-box, AE-box, TCT-box, GATA-box, ATC-motif and GT1-motif. We also identified a large amount of defense and stress response elements, including W-box, Box-s, MBS, Myc, Myb and TC-rich (Figure 4 and Table S4).

### 3.6. Expression Profiles of Putative Cucumber TCP Genes in Different Tissues

**Figure 5:**
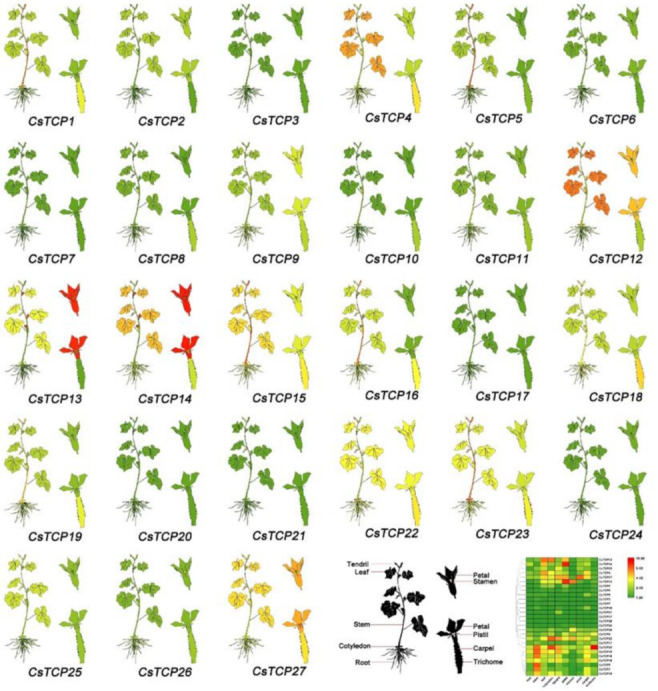
The differential expression of *CsTCPs* in different organs by qRT-PCR. The mean expression values were visualized by Tbtools; Red represents high expression level and green represent low expression level. The relative expression values and standard errors is provided in Table S5.

We analyzed the expression of the 27 putative cucumber *TCP* genes (*CsTCP1-CsTCP27*) in different cucumber organs, including root, stem, leaf, cotyledon, tendril, and flower (petal, stamen, pistil, carpel, pericarp and trichome) by qRT-PCR (Figure 5, Table S5). The results showed that the *CsTCPs* presented distinct expression profiles in different organs, suggesting functional divergence of *CsTCPs* for plant development. The expression levels including *CsTCP22* and *CsTCP23* were consistently high in every organ. In addition, there were seven genes which have high expression level in most organs, including *CsTCP4*, *CsTCP13*, *CsTCP15*, *CsTCP16*, *CsTCP18*, *CsTCP19* and *CsTCP27.* The expression level of these genes was even much higher than *CsTCP22* and *CsTCP23* in specific organs. In contrast, *CsTCP7*, *CsTCP17*, *CsTCP20*, *CsTCP21* and *CsTCP24* were not detected in most organs. Similarly, *CsTCP3, CsTCP6, CsTCP7 and CsTCP8* were also expressed at a very low level in every organ. Interestingly, several low-expressed genes were found to express in specific tissue as follows: *CsTCP10* had the strongest expression in carpel, and *CsTCP21* showed specific expression had the strongest expression in tendril. These genes may play a role in the development of the corresponding phenotype. In addition, we found that *CsTCPs* had low expression level in root. This implied that *CsTCPs* mainly involved in the development of other organs rather than root in cucumber.

### 3.7. Expression Analysis of CsTCPs during Flower Bud Development

**Figure 6:**
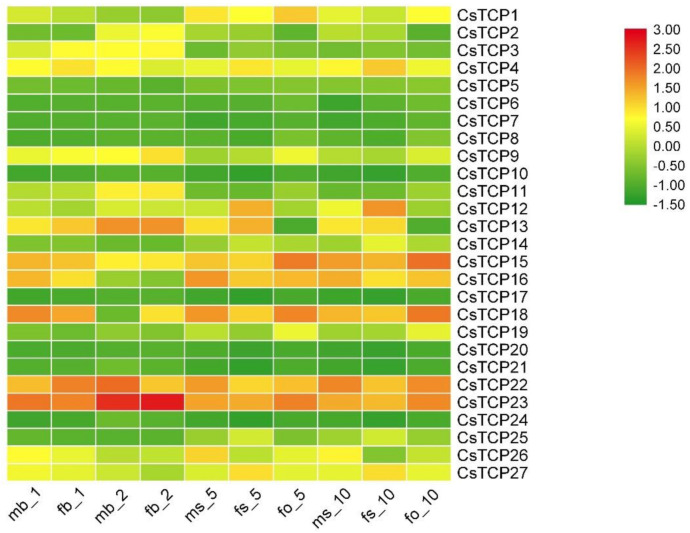
Expression analysis of *CsTCPs* in the development of female and male flower buds. The following abbreviations are used: mb_1, male bud 1 mm; fb-1, female bud 1 mm; fb_2, female bud 2 mm; ms_5, male stamen 5 mm; fs_5, female stigma 5 mm; fo_5, female ovary 5 mm; ms_10, male stamen 10 mm; fs_10, female stigma 10 mm; fo_10, female ovary 10 mm. Genes highly expressed in organs are colored red, and genes lower expressed in organs are colored green.

To elucidate their roles in flower development, the qRT-PCR were conducted to confirm the relative expression levels of 27 *CsTCPs* at different stages of flower bud development (Figure 6). The whole floral development process was divided into 12 stages (s1–s12) The development of female and male flowers were not morphologically distinguishable at s1–s5 (floral bud side: 0.1–0.55 mm). The carpel primordia had just initiated in the female flower at s6 (floral bud side: 0.65 mm), which is the key stage for morphologic divergence from normal to inappropriate organs [41]. Most *CsTCPs* were detected during the flower bud differentiation in cucumber line 9930 except *CsTCP17* and *CsTCP24*, suggesting that most of them may regulate the flower development.

When the length of floral buds was 1 mm, there is no difference in the expression among *CsTCPs* between female and male flower buds. However, *CsTCP4*, *CsTCP12*, *CsTCP13*, *CsTCP14*, *CsTCP25,* and *CsTCP27*, all belong to the CIN subclass, were expressed highly in stigma in the process of female bud differentiation (female flower bud side: 2–10 mm) (Figure 6). Then, the expression of these genes increased with the development of the stigma, implying that these genes were involved in development of female floral organs. Besides, *CsTCP15*, *CsTCP16*, *CsTCP18*, *CsTCP22* and *CsTCP23* were always highly expressed in the early stage of female and male flower bud development. We could not detect the expression of *CsTCP10*, *CsTCP17*, *CsTCP20*, *CsTCP21* and *CsTCP24* in female and male buds with the length of floral organs increasing, implying that these genes should not be involved in the development of floral organs.

### 3.8. Expression Analysis of CsTCPs Under Hormone Treatments and Environmental Stresses

**Figure 7:**
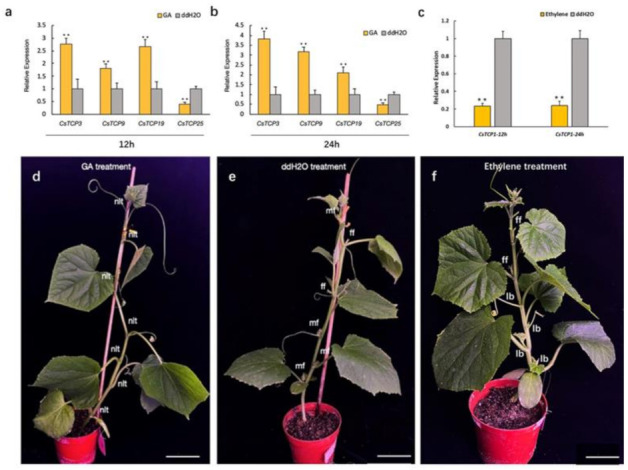
The Morphology and *CsTCPs* expression levels of cucumber plants under gibberellin (GA) and ethylene treatment. (**a**,**b**) Expression analysis of *CsTCPs* at 12 h (**a**) and 24 h (**b**) after GA treatment, respectively. (**c**) Expression analysis of *CsTCP1* at 12 h and 24 h after ethylene treatment. The *y* axis is the scale of the relative transcript abundance level. Error bars represent the standard deviations from three biological replicates. ** stands for significant difference (*p* < 0.01). (**d**–**f**) Morphology of plants after GA treatment (**d**), ddH_2_O treatment (**e**), and ethylene treatment (**f**). nlt, non-lateral tissues; mf, male flower; ff, female flower; lb, lateral branch.

To demonstrate whether *CsTCPs* are involved in the pathway of hormone, we analyzed the expression of *CsTCPs* after GA and ethylene treatment. As shown in Figure 7, *CsTCP*3, *CsTCP9*, *CsTCP19* were up-regulated after 12 h of GA treatment (Figure 7a). The expression level of *CsTCP3* and *CsTCP9* increased, while expression level of *CsTCP19* decreased slightly after 24 h of GA treatment (Figure 7b). However, *CsTCP25* was down-regulated and always maintained at a low level until 24 h (Figure 7a,b). Half a month after GA treatment, we found that the lateral organs, including male, female flower buds and lateral branches, were inhibited (Figure 7d,e and Figure S3). Previous studies showed that *CsTCP3* inhibited axillary bud outgrowth [29]. We considered that up-regulated expression of *CsTCP3* induced by GA treatment should suppress the lateral branch development in cucumber. After 12–24 h of ethylene treatment, the expression of most *CsTCPs* were not changed significantly, except *CsTCP1*. The expression of *CsTCP1* was down-regulated and maintained at a low level (Figure 7c). Half a month after ethylene treatment, the number of lateral branches was increasing and the lateral branches were growing at the low number of nodes of plants (Figure 7e,f and Figure S3). The above results indicated that these *CsTCPs* may participate in the development of lateral organs in response to GA and ethylene in cucumber.

**Figure 8:**
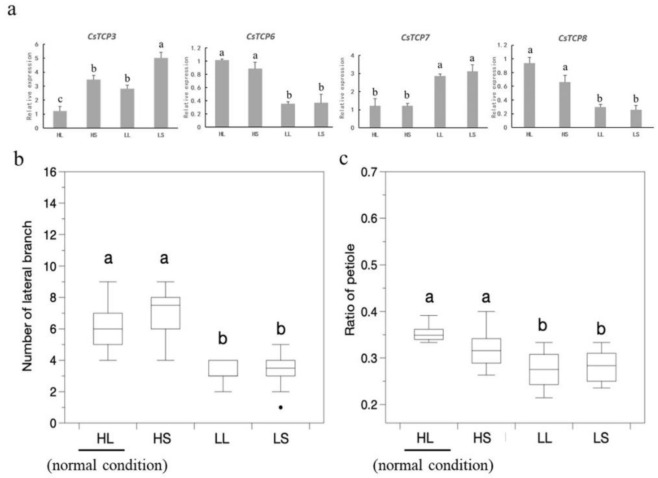
Phenotypes and *CsTCPs* expression levels of cucumber plants under different temperature and photoperiod treatments. (**a**) Expression analysis of four *CsTCPs* under different temperature and photoperiod treatment. (**b**) Comparison of the number of lateral branch and (**c**) Ratio of the petiole of cucumber under HL, HS, LL and LS condition. HL, high temperature and long day, as the normal condition; HS, high temperature and short day; LL, low temperature and long day; LS, low temperature and short day. Error bars represent the standard deviations from three biological replicates. Different letters indicate significant difference.

To understand the functions of *CsTCPs* in the stress-related environmental adaptation, we analyzed the expression of *CsTCPs* and the phenotype of cucumber seedings treated with temperature and photoperiod. As shown in Figure 8, after different temperature and photoperiod treatments, the trend of the expression of *CsTCP6* and *CsTCP8* were similar. Compared with the normal condition, the expression of *CsTCP6* and *CsTCP8* decreased under LL and LS condition However, the *CsTCP7* was up-regulated under LL and LS condition (Figure 8a). Additionally, *CsTCP3* was induced significantly under LS condition, and was up-regulated slightly under LL and HS condition (Figure 8a). Compared with the normal condition, the number of lateral branches of the cucumber was significantly reduced, and the ratio of petiole of plant was also decreased under LL and LS condition (Figure 8b,c). These results indicated that *CsTCPs* may participate in the development of lateral organs in response to temperature and photoperiod in cucumber.

## 4. Discussion

Plant-specific transcription factors TCPs play various roles in multiple aspects of plant growth and development. In different species, the general organization of TCP family is conserved, and most of them are divided into two classes [9,11,42]. In cucumber genome, we identified 27 putative *CsTCP* genes, more than the previous study that identified 22 *CsTCPs* [30]. Phylogenetic analysis showed the 27 CsTCPs were divided into two classes, Class I and Class II. This result is consistent with the previous classification [6]. By analyzing the distribution of motifs, we found Motifs 4 and 5 are observed in most Class I members, and the great majority of members in the class I included Motif 2. Thus, the CsTCPs in the same class had similar motif composition. Similarly, miR319 binding sites analysis of *CsTCPs* had similar results. In cucumber, we identified four putative miR319-targeted *TCP* genes including *CsTCP12*, *CsTCP14*, *CsTCP25* and *CsTCP27*, all of which belonged to CIN subclass. In general, genes in the same class shared the similar gene structure and motif distribution. However, the gene structure of *CsTCPs* in the same class had no obvious regularity. The reason may be that most putative *CsTCP* genes structure was relatively simple.

Most of *CsTCPs* in Class I had high expression level in every organ, except *CsTCP24*. *CsTCP1*, *CsTCP5* and *CsTCP19* were closely associated with *Arabidopsis AtTCP14* (*At3G47620*) and *AtTCP15* (*At1G69690*) (Figure S2). *CsTCP1*, *CsTCP5* and *CsTCP19* had the strongest expression in the stem. The *AtTCP14* and *AtTCP15* were involved in regulating the internode elongation and trichome branching in *Arabidopsis* [43,44]. *CsTCP4* and *CsTCP27* were highly expressed in carpel and pistil, respectively, and both maintained high expression levels in stigma during the female flower development. *AtTCP2*, an Arabidopsis ortholog of *CsTCP4* and *CsTCP27* was most strongly expressed in flowers, but not other organs [9]. These results suggest these genes may have a similar function in different species.

In CYC/TB1 subclass, most members were expressed at a very low level in all organs, especially *CsTCP3*, *CsTCP7*, *CsTCP17* and *CsTCP20* were not detected in almost organs. In addition, we found that some genes in CYC/TB1 subclass were expressed in specific organs. *CsTCP10* showed specific expression in carpel, *CsTCP21* had the specific expression in tendril. However, the *Arabidopsis* homolog of *CsTCP10* and *CsTCP21* is *AtTCP1* (*At1G67260*), whose function is to regulate the elongation of leaves [45]. The *CsTCPs* presented distinct expression profiles in various organs, suggesting their functional divergences during cucumber development.

Previous studies have shown that *TCP* genes play a key role in flower development. *PeCIN8* is involved in the regulation of ovule development in *Phalaenopsis equestris* [12]. In *Prunus mume,* the CIN type genes were highly expressed in ovule development stages and pistil initiation [39]. In this study, *CsTCP12*, *CsTCP13*, *CsTCP14* and *CsTCP25* all belong to the CIN subclass, which were also expressed highly in stigma, and their expressions increased with the development of the stigma. The result implied these CIN type genes play an important role in development of female floral organs in cucumber. The Class I type genes, *CsTCP15*, *CsTCP16*, *CsTCP18*, *CsTCP22* and *CsTCP23* were always highly expressed in the male and female flower bud development. Class I type gene in *Chrysanthemum morifolium, CmTCP14* suppresses organ size and prolong flowering time [46]. In *Arabidopsis*, *AtTCP11 (At2G37000)* and *AtTCP16 (At3G45150)* participate in early pollen development [47,48]. These results suggested that CIN type and Class I type *CsTCP*s may be involved in the flower development of cucumber.

GA and ethylene are the key endogenous regulator of plant development and growth [49,50]. In our study, we found that the growth of lateral organs was inhibited after GA treatment, and the lateral branches grew at the low nodes of plants after ethylene treatment. The *cis*-acting elements analysis of 27 *CsTCPs* showed that several GA-related *cis*-elements and ERE elements in their promotors were identified. The expression of some *CsTCPs* were significantly changed after GA treatment, including *CsTCP3* and *CsTCP19*. However, although the promotors of *CsTCP9* and *CsTCP25* contain no GA-related *cis*-elements, the expression of *CsTCP9* and *CsTCP25* was up-regulated and down-regulated after GA treatment, respectively. These results suggested that GA affects the development of lateral organs by directly or indirectly regulating the expression of *CsTCPs*. The pea PsBRC1 acted as a typical branch number regulator, involving in GA signal [20]. Although many *CsTCPs* contain ERE elements, ethylene treatment did not affect the expression of these genes. While *CsTCP1* contains no ERE elements, its expression was significant decreased and maintained at low level after ethylene treatment. These results indicated that *CsTCP1* should regulate the lateral branch development by participating in ethylene signal pathway. Motif 1 was presented in almost CsTCPs except CsTCP 1. CsTCP1 lacking Motif 1 may have acquired new functions during evolution in cucumber.

Environment conditions show pronounced impacts on the shoot branching habit of plant [51,52]. In this study, we found that the number of lateral branches was increasing under shorter photoperiod and low temperature, and four *CsTCPs* were induced by the environment changes. It demonstrated that the four putative *CsTCP* genes may participate in the lateral branch development by responding to temperature signals. The *CsTCP3* was induced by the low temperature and short photoperiod, and it was also induced by GA treatment. Previous studies suggested that *CsTCP3* suppresses the lateral bud growth by controlling the accumulation of auxin in cucumber axillary buds [29]. We found that both of *CsTCP3* (*Csa1G020890.1*) and *CsTCP7* (*Csa1G042180.1*) were closely associated with *Arabidopsis AtTCP18* (*BRC1*). *AtTCP18* plays a vital role in the control of shoot branching [39]. Hence, we speculated that *CsTCP3* may regulate lateral branching development by participating in multiple signaling pathways.

## Supplementary Materials

The following are available online at https://www.mdpi.com/2073-4425/11/11/1379/s1, Table S1: Primers for qRT-PCR in this study, Table S2: The basic information of 27 putative TCP genes, Table S3: Collinear relationships of TCP family members in the cucumber and between the melon and *Arabidopsis*, Table S4: Cis-acting elements on promotors of CsTCPs, Table S5: Expression levels of cucumber TCP genes in different tissues, Figure S1: Physical locations of putative TCP genes on cucumber chromosomes. Scale bar on the left represents the length of the chromosome(bp), Figure S2: Phylogenetic tree of TCPs in Arabidopsis and cucumber. The phylogenetic tree was constructed based on TCPs sequences from cucumber (27 proteins, marked with black dot) and Arabidopsis (24 proteins). A specific color indicates each of the three classes, Figure S3: Comparison the number of lateral tissues of cucumber under GA and ethylene treatments. Error bars represent the standard deviations from three biological replicates (** < 0.01, Student’s *t* test).
