# Supplementary figures and images for: Genome-Wide Identification and Characterization of the TCP Gene Family in Cucumber (Cucumis sativus L.) and Their Transcriptional Responses to Different Treatments
Source: Genes (Basel). 2020 Nov 20;11(11):1379. doi: 10.3390/genes11111379 (PMC7709023; doi:10.3390/genes11111379)

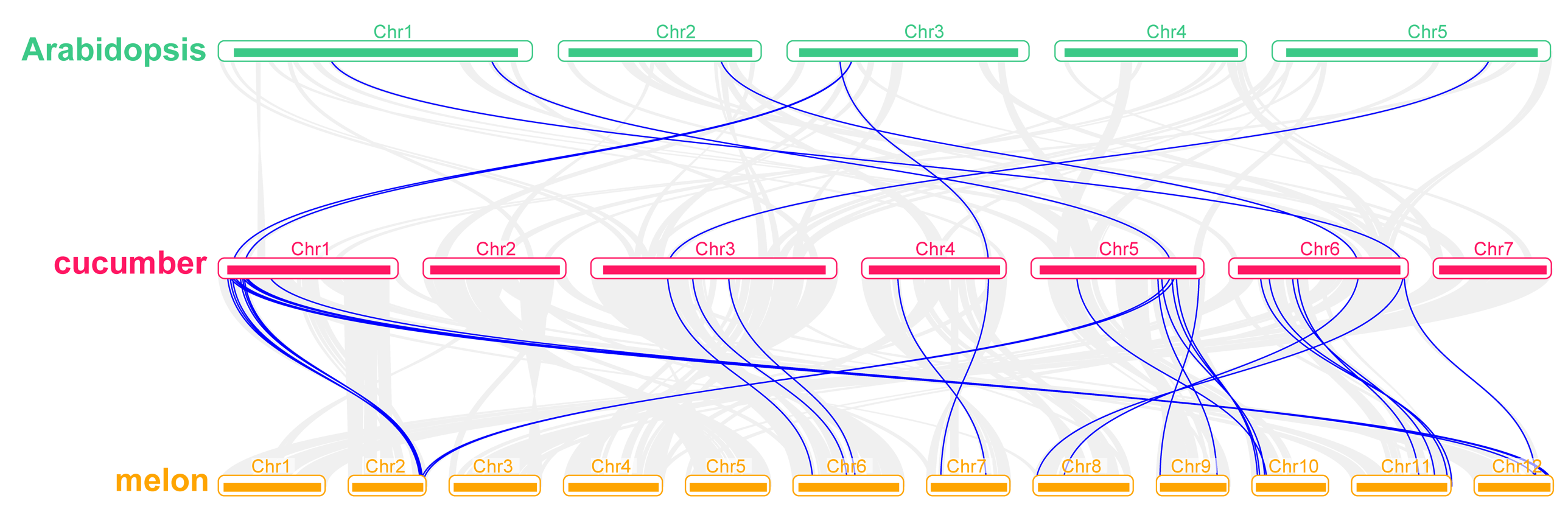

Supplement: Supplementary file 1 [file genes-11-01379-s001.zip › Figure and supplementary files/Figure/Figure 2. Collinear relationships of genes pairs from cucumber, melon and Arabidopsis.jpg]

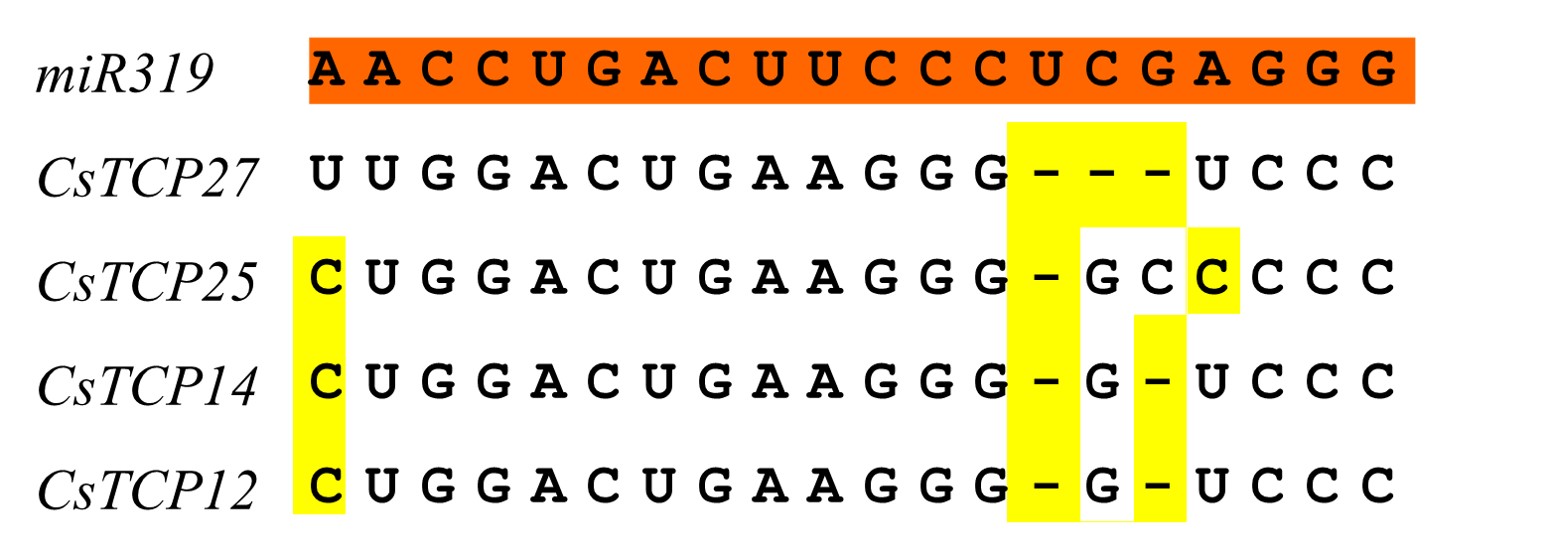

Supplement: Supplementary file 1 [file genes-11-01379-s001.zip › Figure and supplementary files/Figure/Figure 3. Alignment of putative target areas for miR319.jpg.jpg]

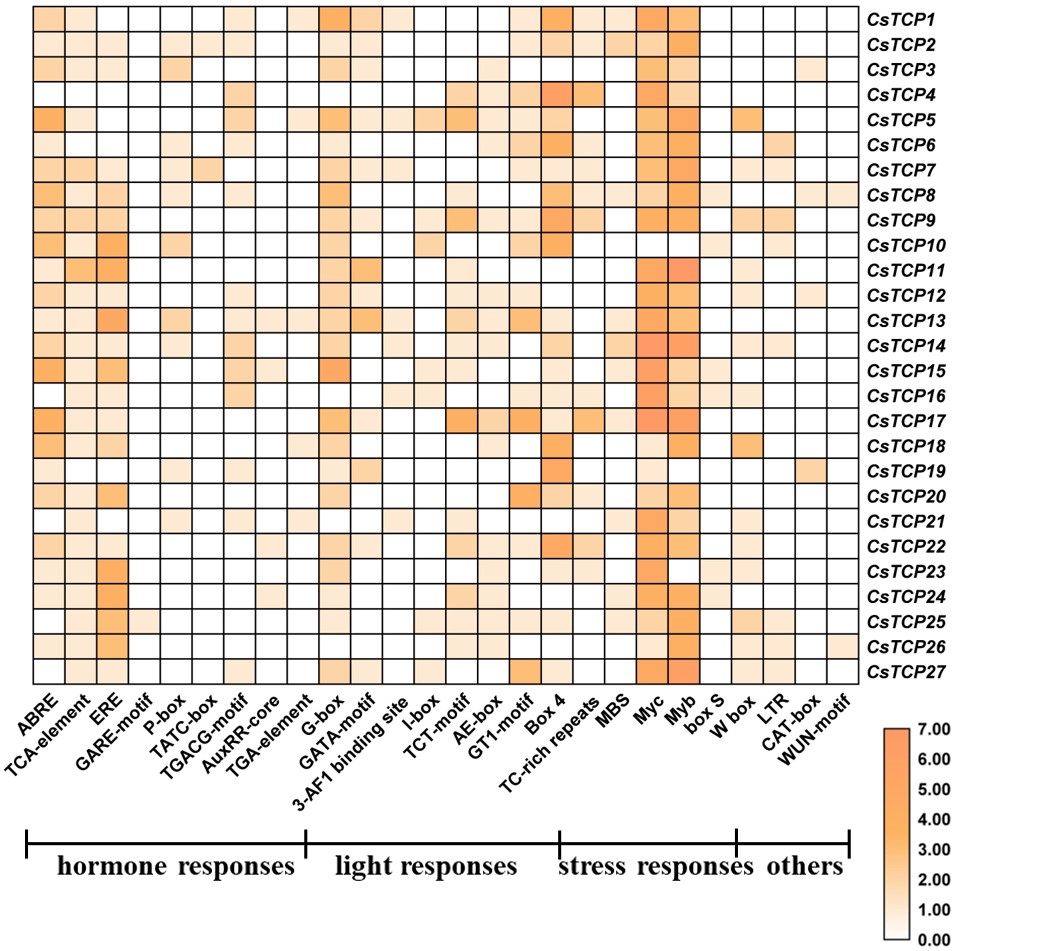

Supplement: Supplementary file 1 [file genes-11-01379-s001.zip › Figure and supplementary files/Figure/Figure 4. Cis-acting elements on promotors of CsTCPs..jpg]

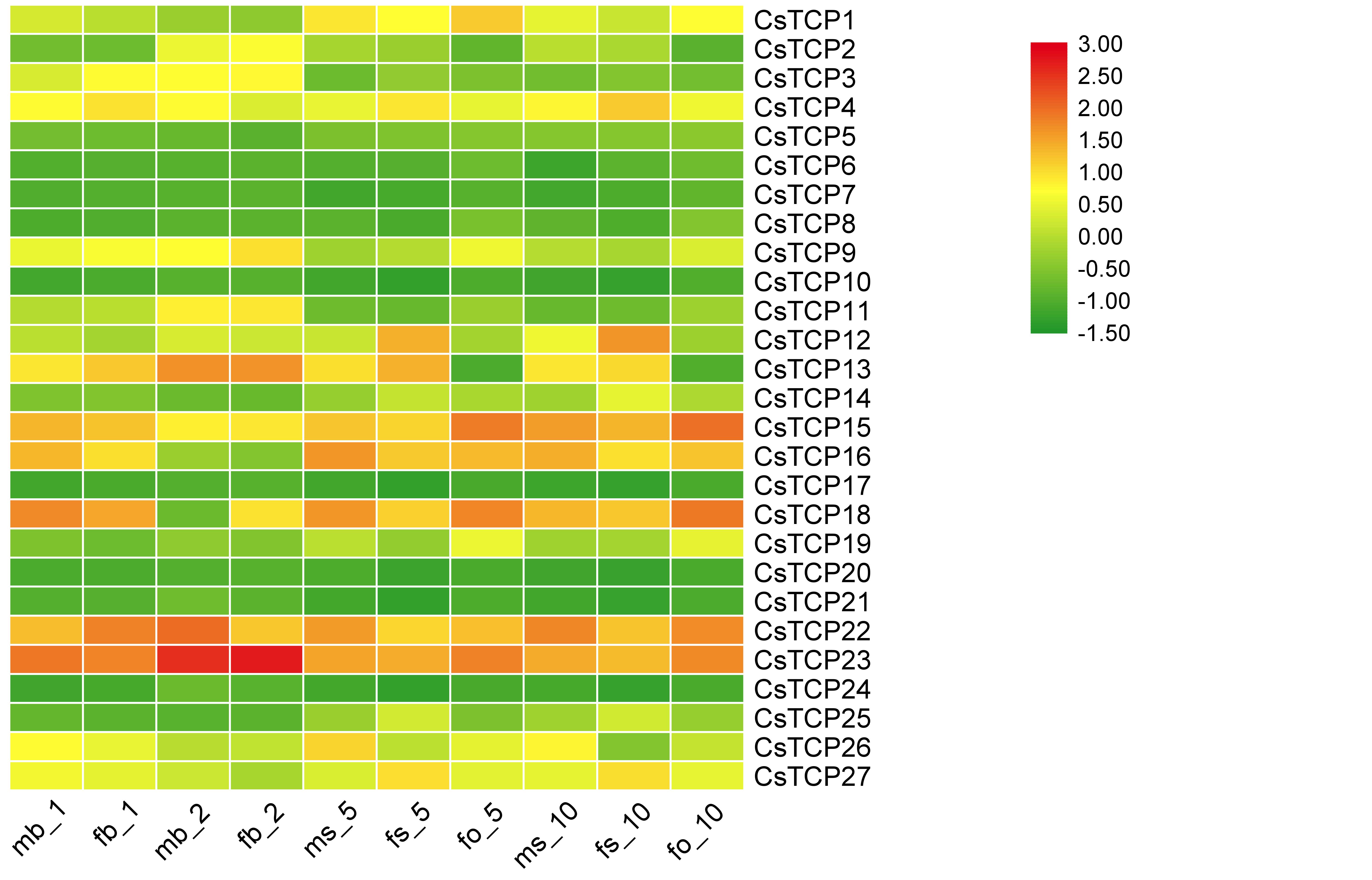

Supplement: Supplementary file 1 [file genes-11-01379-s001.zip › Figure and supplementary files/Figure/Figure 6. Expression analysis of CsTCPs in the development of female and male fl.jpg]

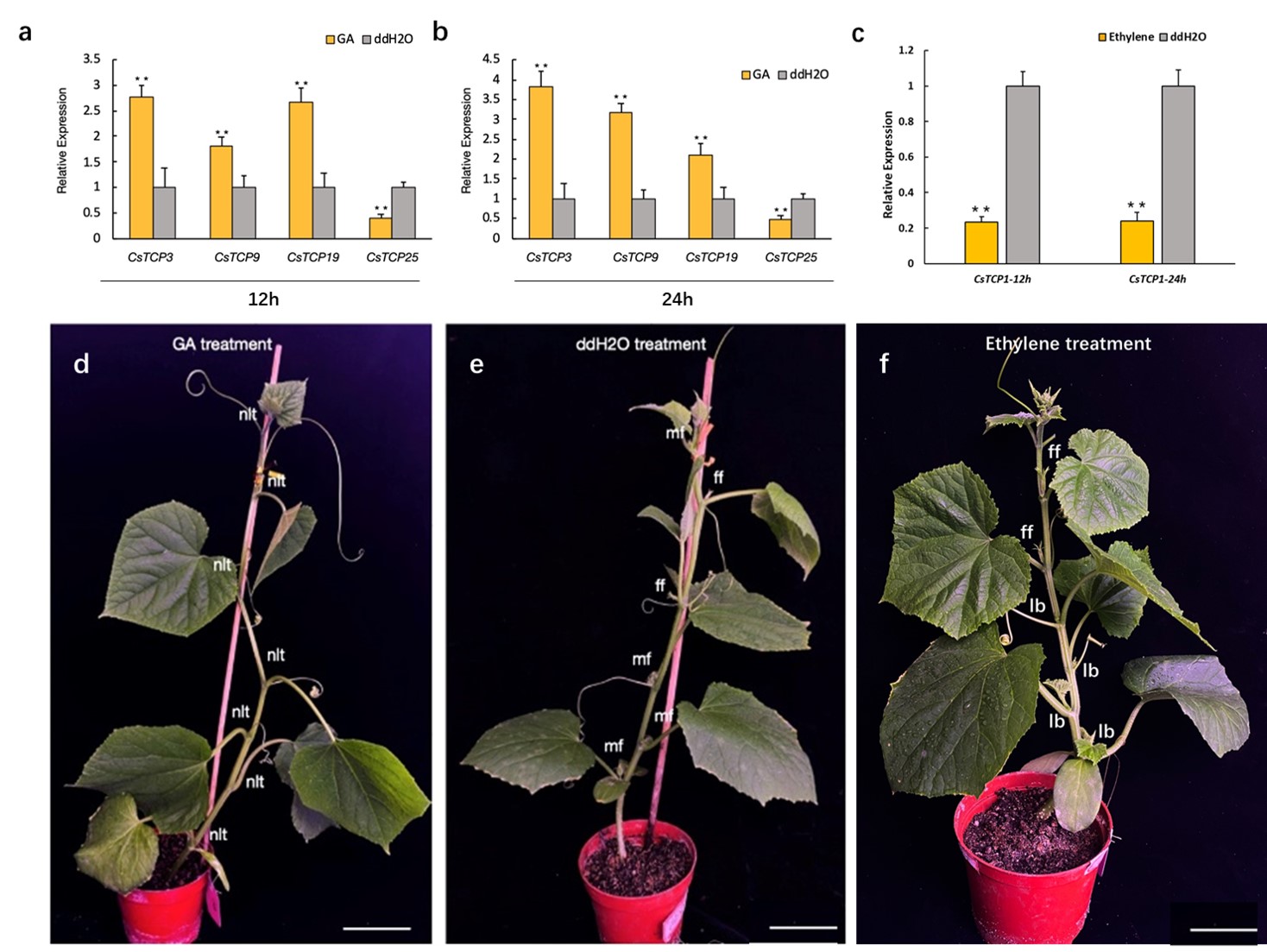

Supplement: Supplementary file 1 [file genes-11-01379-s001.zip › Figure and supplementary files/Figure/Figure 7. The Morphology and CsTCPs expression levels of cucumber plants under GA and ethylene treatment.jpg]

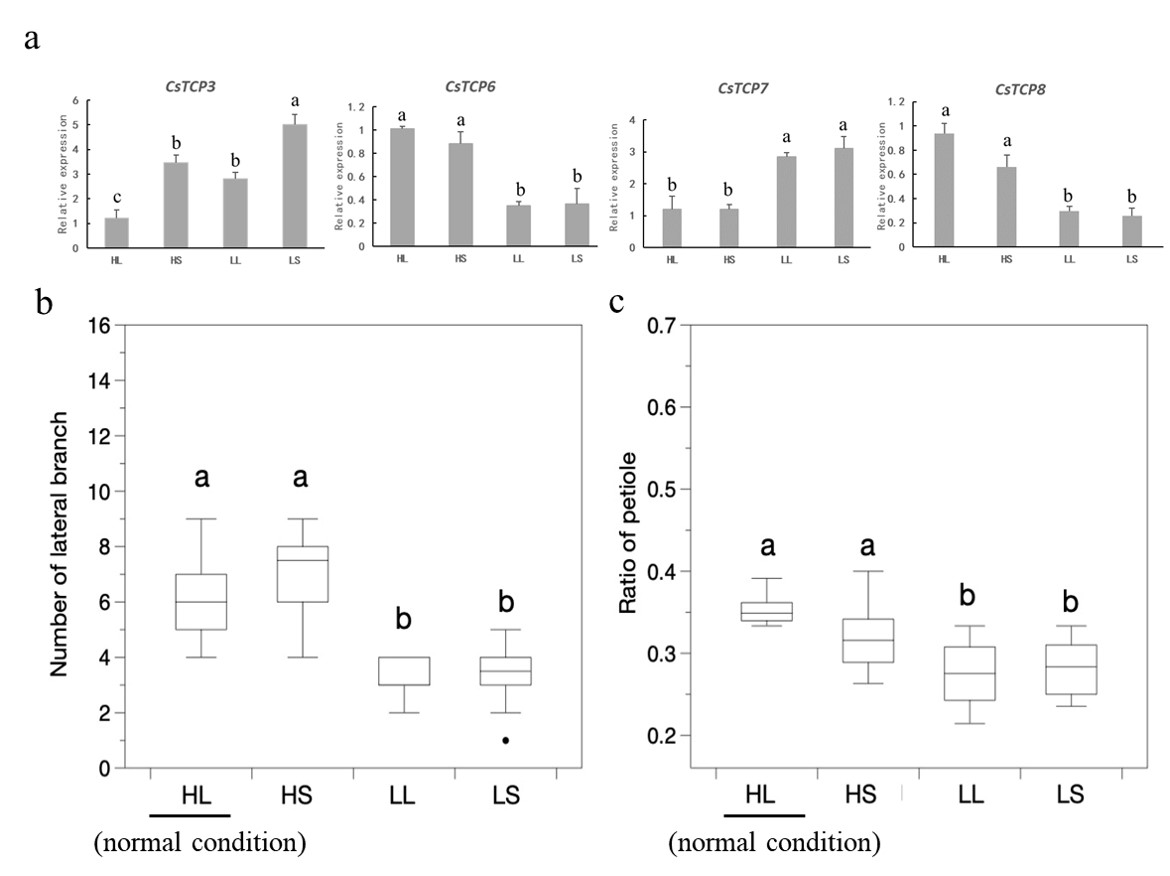

Supplement: Supplementary file 1 [file genes-11-01379-s001.zip › Figure and supplementary files/Figure/Figure 8. Phenotypes and CsTCPs expression levels of cucumber plants under different temperature and photoperiod treatments.jpg]

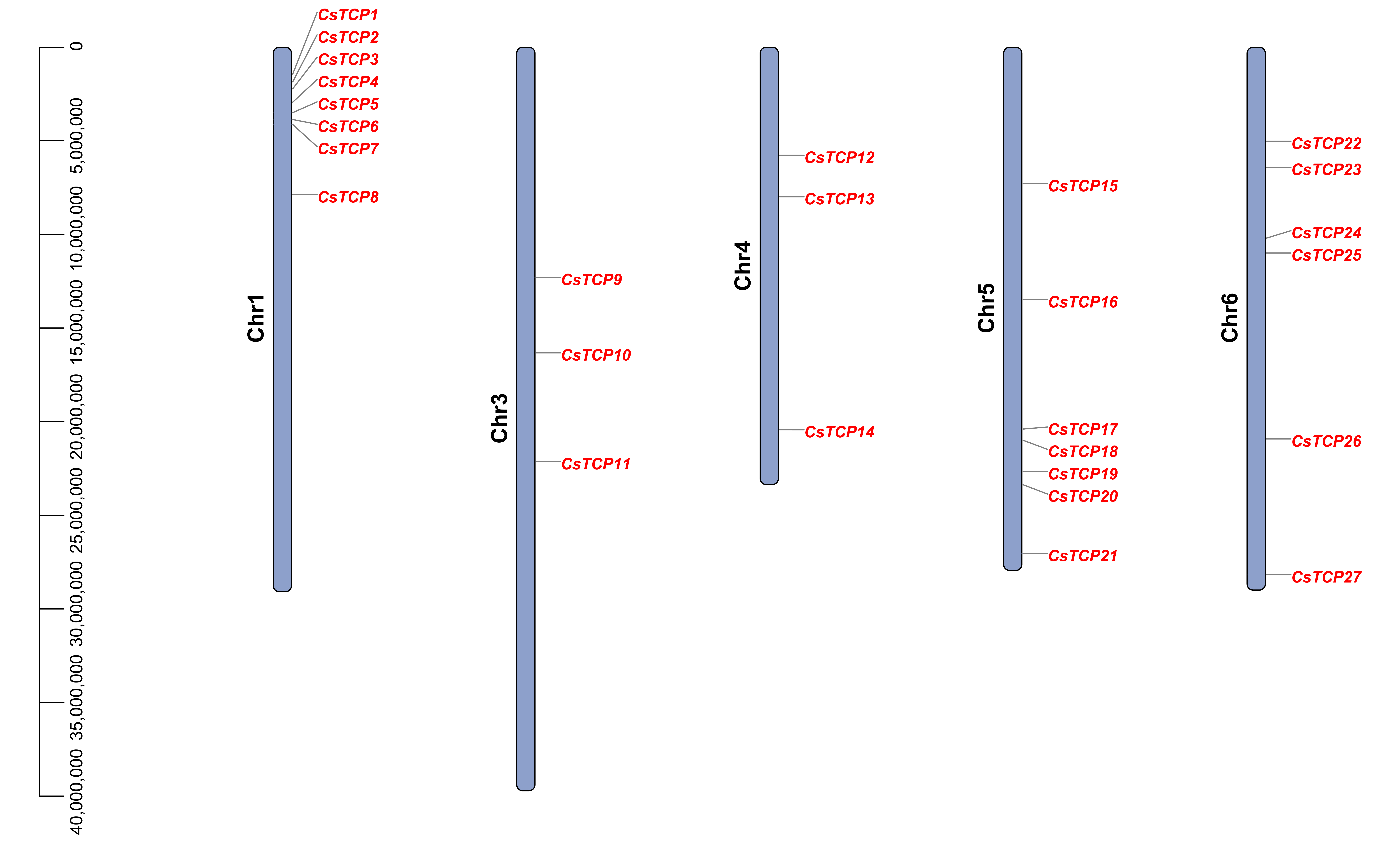

Supplement: Supplementary file 1 [file genes-11-01379-s001.zip › Figure and supplementary files/supplementary files/Figure S1. Physical locations of TCP genes on cucumber chromosomes..jpg]

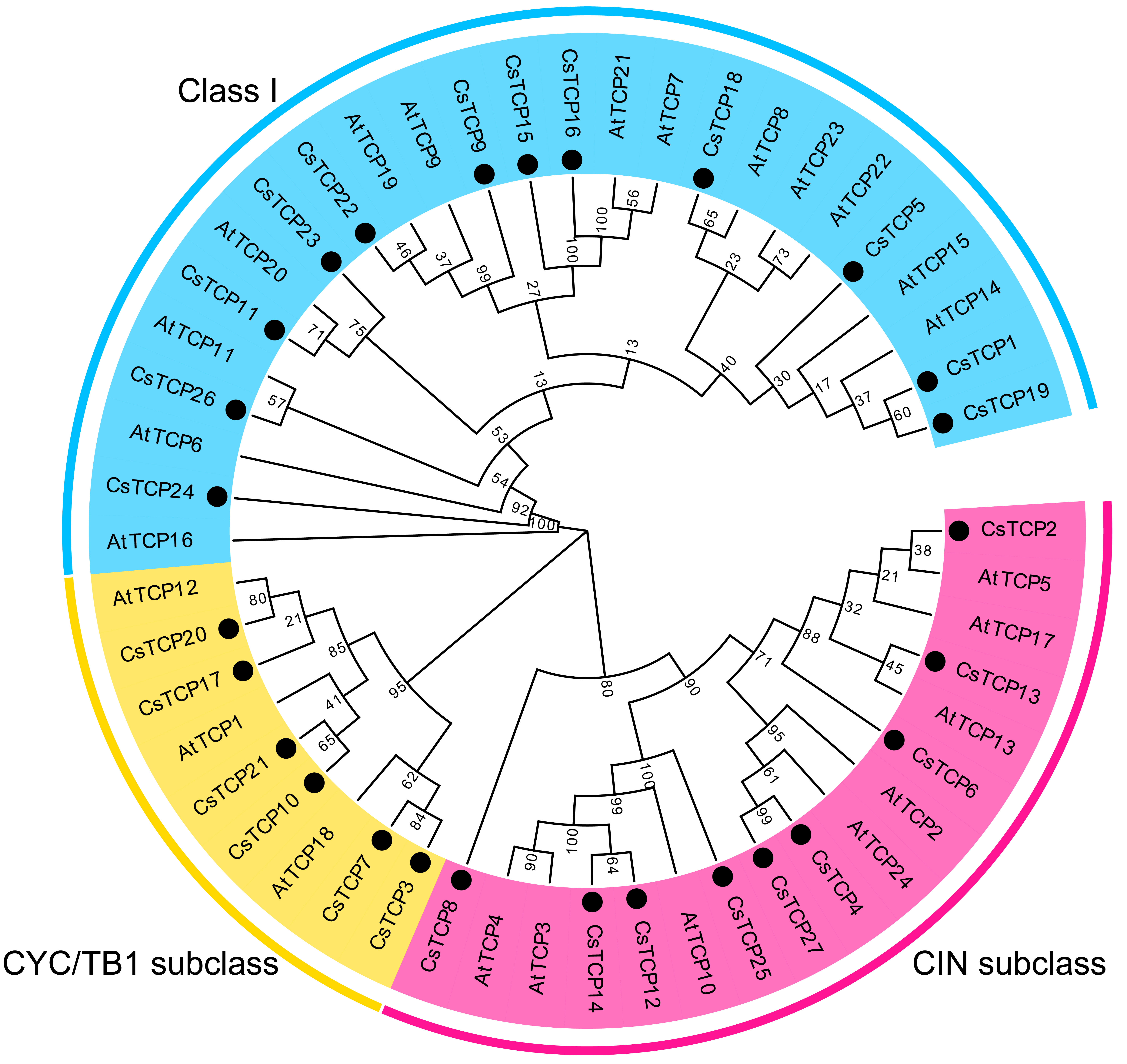

Supplement: Supplementary file 1 [file genes-11-01379-s001.zip › Figure and supplementary files/supplementary files/Figure S2. Phylogenetic tree of TCPs in Arabidopsis and cucumber.jpg]

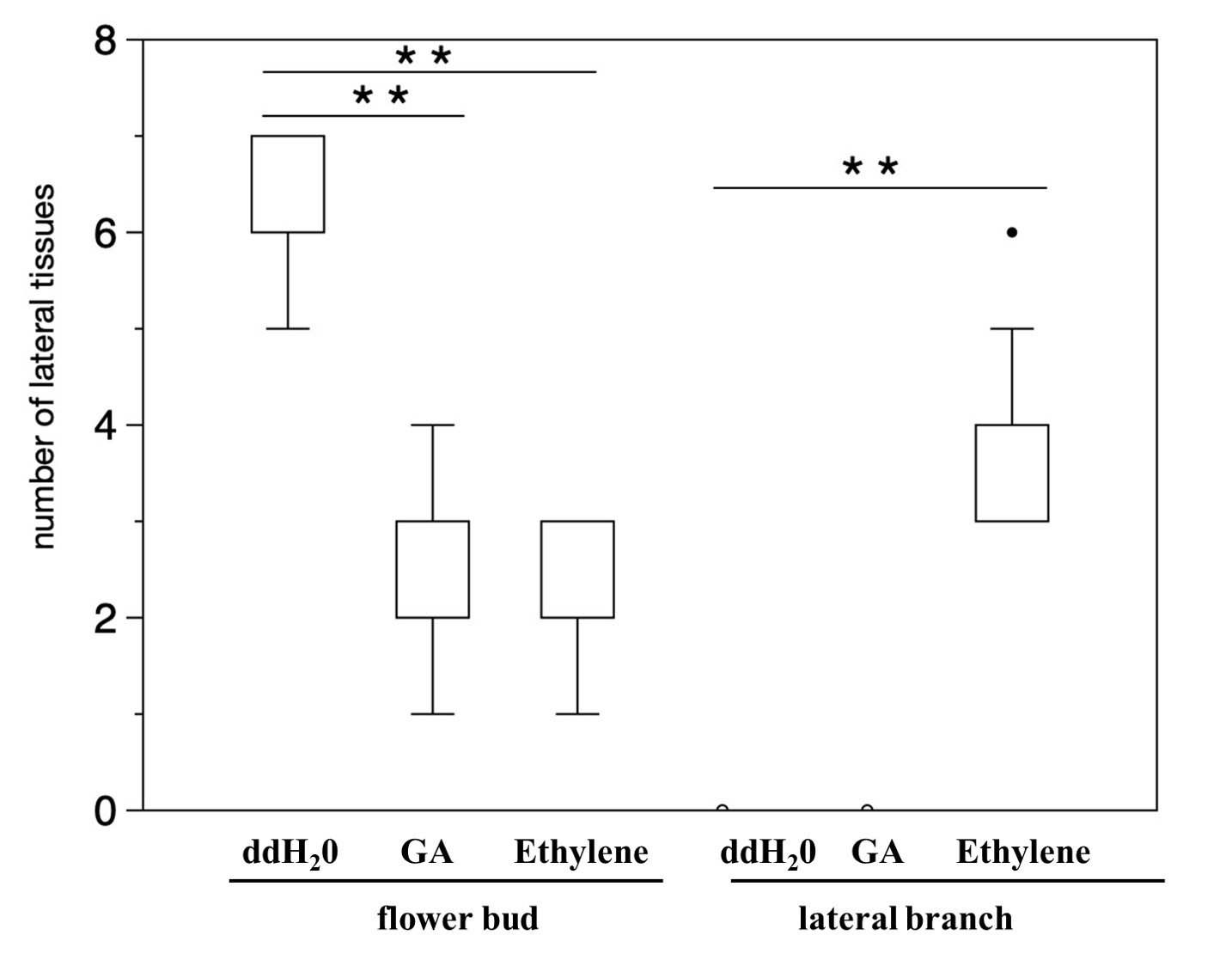

Supplement: Supplementary file 1 [file genes-11-01379-s001.zip › Figure and supplementary files/supplementary files/Figure S3.Comparison the number of lateral tissues of cucumber under GA and ethylene treatments..jpg]
